# Fish Scale for Wearable, Self-Powered TENG

**DOI:** 10.3390/nano14050463

**Published:** 2024-03-03

**Authors:** Liwei Zhao, Jin Han, Xing Zhang, Chunchang Wang

**Affiliations:** Laboratory of Dielectric Functional Materials, School of Materials Science & Engineering, Anhui University, Hefei 230601, China; ms22301080@stu.ahu.edu.cn (L.Z.); b21301143@stu.ahu.edu.cn (J.H.); ms22201002@stu.ahu.edu.cn (X.Z.)

**Keywords:** fish scale, hydrogen bond, collagen, triboelectric effect, triboelectric nanogenerators

## Abstract

Flexible and wearable devices are attracting more and more attention. Herein, we propose a self-powered triboelectric nanogenerator based on the triboelectric effect of fish scales. As the pressure on the nanogenerator increases, the output voltage of the triboelectric nanogenerator increases. The nanogenerator can output a voltage of 7.4 V and a short-circuit current of 0.18 μA under a pressure of 50 N. The triboelectric effect of fish scales was argued to be related to the lamellar structure composed of collagen fiber bundles. The nanogenerator prepared by fish scales can sensitively perceive human activities such as walking, finger tapping, and elbow bending. Moreover, fish scales are a biomass material with good biocompatibility with the body. The fish-scale nanogenerator is a kind of flexible, wearable, and self-powered triboelectric nanogenerator showing great prospects in healthcare and body information monitoring.

## 1. Introduction

The rapidly growing demands for healthcare and body information monitoring have spurred great interest in flexible and wearable energy devices [1]. Due to the constant use of traditional energy sources, our environmental problems are becoming more and more serious, and there is an urgent need for new energy sources to replace traditional energy sources. Wind, biomass, geothermal, hydro, and ocean energy are some of the energy sources that can replace traditional energy sources, but they are not readily available. Thus, we should look for a new source of energy that can be used at any time. Friction nanogenerators are a very suitable energy source due to their high power density. Among them, flexible triboelectric nanogenerators (TENGs) play a vital role in healthcare. For example, Chen et al. proposed a TENG with tile nanostructures for the detection of human health with an optimal output voltage of 37.8 V, an output current of 1.8 μA, and a transmitted charge of 14.1 nC, which can be very sensitive for the detection of human health [2]. According to the conversion mechanism, TENGs are generally classified into four modes, which are the vertical contact-detachment mode [3,4,5,6], horizontal sliding mode [7,8,9,10], single-electrode mode [11,12,13,14] and independent layer mode [14,15,16,17,18].

Harvesting energy from the environment and converting it into electricity allow the energy resources in the environment to be fully utilized to extend the life of batteries or to power electronic devices. In 2006, Wang’s team converted mechanical energy from the environment into electrical energy through the piezoelectric properties in zinc oxide, which could power nanodevices, which Wang’s team called piezoelectric nanogenerators [19]. In 2012, Wang’s team discovered another new device through a polymer film, which Wang’s team named a friction nanogenerator, a device that uses mechanical deformation to achieve electrical energy output [20]. The TENG is used to power the device by collecting ambient energy and converting it into electrical energy, or is used as a sensor. Han et al. fabricated a TENG based on the contact separation mode by doping chitosan and carbon nanotubes. They used the chitosan–carbon nanotube composite as the positive electrode material and fluorinated ethylene propylene as the negative electrode material. The optimal performance of their TENG showed an open-circuit voltage of 85.8 V, a short-circuit current of 8.7 μA, and a transmitted charge of 29 nC, which could be used to detect the human body’s activities well [21]. Inspired by the fish scale structure, Ma et al. designed a TENG based on a symmetrical arrangement of the fish scale structure, which can be used to collect the energy due to sliding and to detect the rotational state. This design provides a new idea for the application of the TENG. In addition, the method is very simple in terms of device structure and also avoids the energy dispersion caused by the use of multiple electrodes [22].

With the development of triboelectric nanogenerators, self-powered triboelectric nanogenerators have been widely used in wearable electronic devices, electronic skin, and medical testing because of their self-powered characteristics. For example, in wearable electronic devices, Liu et al. proposed a polymer membrane to fabricate a TENG with a nano-wrinkled flexible braided structure, which was designed to be comparable to a TENG with an optimal braided structure and whose output signal could last for a long time due to its long-term stability. It works well as a wearable electronic device for collecting human energy, human–computer interaction, and pressure sensing [23]. Chen’s team designed a material with different hydrophilicity along the thickness direction through electrostatic spinning/electrospray technology, which exhibited hydrophilicity on the side close to the skin, and, thus, providing a good solution to the effects at the interface between the e-skin and the skin. This work presents a new approach to designing electronic skin that can also be used to detect human health [24]. Liu et al. proposed a self-powered sensor for detecting the wear and tear of human prosthetic joints, which could be used to detect debris from prosthetic wear and tear. This work promotes the development of the TENG in medical detection [25]. Triboelectric effect materials include electropositive and electronegative materials [26,27,28,29]. To meet the needs of miniaturization, along with being portable, flexible, and wearable, with low-power consumption, more and more self-powered nanogenerators are made based on ceramic–polymer composites and biomaterials. For example, a triboelectric–piezoelectric hybrid nanogenerator with a self-charging pumping system based on BaTiO_3_-Nanorods/Chitosan was found to show enhanced output performance [30]. The use of natural materials is preferable to the use of traditional materials because natural materials are the most compatible with man and nature and are generally more biocompatible, and the use of natural materials in the preparation of TENGs makes better use of resources. Natural biomass materials, such as fish scales [31], chitosan [32], gelatin [33], spider silk [34], onion skins [35], etc., were reported to be widely used in nanogenerators or biosensors. Singh et al. used the scales of Rohu fish to fabricate a TENG based on the contact separation mode. Different materials were used as cathode materials, comparing the position of the fish scales on the friction electric series [31]. Yar et al. proposed a chitosan/clay TENG and investigated the effect of the type of clay on the TENG, providing more possibilities for the choice of materials for the TENG and its ability to elicit an appropriate response at specific sites in the body [32]. Pan et al. present a fully biodegradable triboelectric nanogenerator based on gelatin film and electrospun polylactic acid nanofiber membrane. The TENG can be used for environmental monitoring and can be completely dissolved without harming the human body [33]. Zhang et al. genetically engineered recombinant spider silk protein (RSSP) to design a biocompatible triboelectric material with programmable triboelectric properties, multiple functionalizations, large-scale manufacturability, and extraordinary output performance [34]. Zhang et al. report a study on the hetero-triboelectric effects (HTEs) of half-cell allium plant skins such as leek, scallion, and onion. Single-material TENGs (SM-TENGs) have been fabricated based on the two surfaces of these plant skins, taking advantage of their HTEs [35]. Recently, Liu et al. proposed a TENG that can be used to monitor vital signs in the cardiovascular system. Its miniaturization and flexibility can reduce the potential damage to cardiac tissues by implantation, giving it tremendous promise for applications in minimally invasive techniques [36]. These studies provide the effective monitoring of human physiological health, highlighting the importance of biocompatible materials in energy collecting [37]. It shows that self-powered nanogenerators play a crucial role in the fields of wearable, motion monitoring, and medical monitoring [38,39]. Chen et al. discuss sensing mechanisms for pulse-to-electricity conversion, analytical models for calculating cardiovascular parameters, and application scenarios for textile TENGs. Chen et al. provide a prospective on the challenges that limit the wider application of this technology and suggest some future research directions. In the future, textile TENGs are expected to make an impact in the fields of wearable pulse wave monitoring and CVD diagnosis [38]. An et al. report such a neck motion detector comprising a self-powered triboelectric sensor. This developed neck motion detector has promising applications in neck monitoring, rehabilitation, and control [39]. However, at present, the manufacturing process of some flexible wearable devices is more troublesome, and some materials are polymer materials that are difficult to be degraded [40], which will lead to the problem of e-waste in the current global pursuit of a green environment [41]. A flexible, wearable TENG for monitoring human activities requires that the sensing materials are flexible and biocompatible [42,43,44,45]. Therefore, we propose a flexible, wearable, self-powered TENG based on the biological waste fish scale. This material is used because fish scales contain a lot of collagen [46], which has been evidenced to be an excellent triboelectric effect material [47]. 

The ability of fish scales as a sensing material for self-powered TENGs and the output signals under different conditions were investigated. Our results showed that the voltage supplied by the TENG can charge a capacitor. Since collagen is widely present in organisms, it is biocompatible with human beings and completely biodegradable. Moreover, many fish scales are thrown away as waste around the world every day, resulting in a huge waste of resources. We believe that fish scales hold great prospects for large-scale applications of flexible wearable TENG. 

## 2. Experiment

### 2.1. Fish Scale Treatment

**Figure 1:**
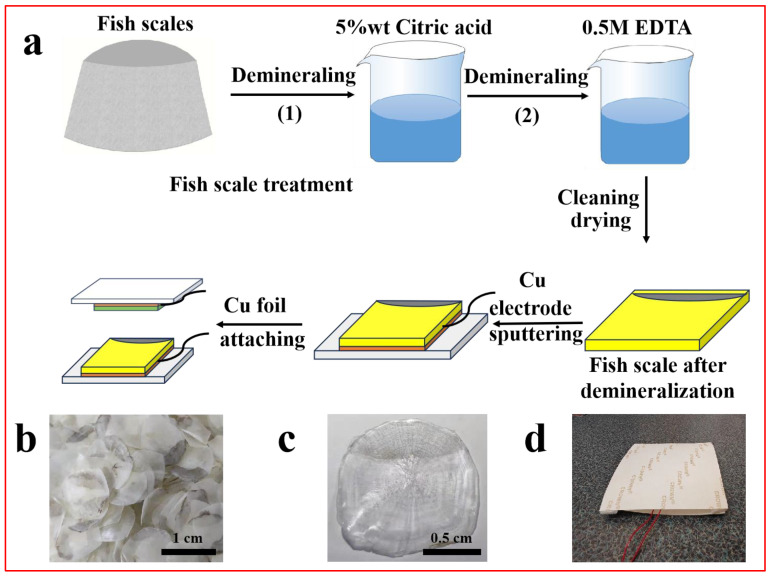
(**a**) The processes of fish scale demineralization and self-powered TENG fabrication. Photos of (**b**) the discarded fish scales, (**c**) a demineralized fish scale, and (**d**) a fish-scale-based TENG.

Fish scales were collected from fresh carp. The fish scale treatment was illustrated in the upper row of Figure 1a. The scales were rinsed with deionized water and dried at 50 °C for 48 h. The cleaned scales were demineralized first by immersing in 5 wt% citric acid solution with a solid–liquid ratio of 1:30 for 4 h and then in 0.5 M ethylenediamine tetraacetic acid (EDTA) solution at a solid–liquid ratio of 1:30 for 4 h. Finally, the demineralized scales were washed with deionized water and dried for TENG fabrication. 

### 2.2. TENG Fabrication

The TENG fabrication process was illustrated in the lower row of Figure 1a. Fish scales were used as electropositive materials and fluorinated ethylene propylene (FEP) as electronegative materials. The contact-separation-type TENGs were fabricated using the decalcified fish scales and FEP film as the friction layer components. Copper foils (2 cm× 2 cm) were implemented for conductive electrodes with each electrode linked to a copper wire for signal collection.

### 2.3. Characterizations

The crystallinity of the demineralized fish scales was examined by X-ray diffraction (XRD, Rigaku Smartlab Beijing Co., Beijing, China) with Cu Kα radiation. The functional groups were analyzed by a Fourier microscope (Vertex 80+ Hyperon 2000, Bruker, Karlsruhe, Germany) and a laser Raman spectrometer (Invia-refle). The surface morphology was observed by a scanning electron microscope (SEM, Regulus 8230, Hitachi Co., Tokyo, Japan). The dielectric and ferroelectric properties of the fish scales were measured by a Wayne Kerr 6500B precise impedance analyzer (Wayne Kerr Electronic Instrument Co., Shenzhen, China) and a ferroelectric hysteresis measurement tester (MultiFerroic II, Radiant technologies Inc., Albuquerque, NM, USA), respectively. The output voltage and current of the fish scale TENG were measured by a Keithley 6517B electrometer (Model 6517B Programmable Electrometer, Keithley, Cleveland, USA). The change in mass of the fish scale is investigated by thermogravimetric analyzers (TGA, TGA5500, TA Instruments, New Castle, USA).

## 3. Results and Discussion

### 3.1. Fish Scale Characterization

**Figure 2:**
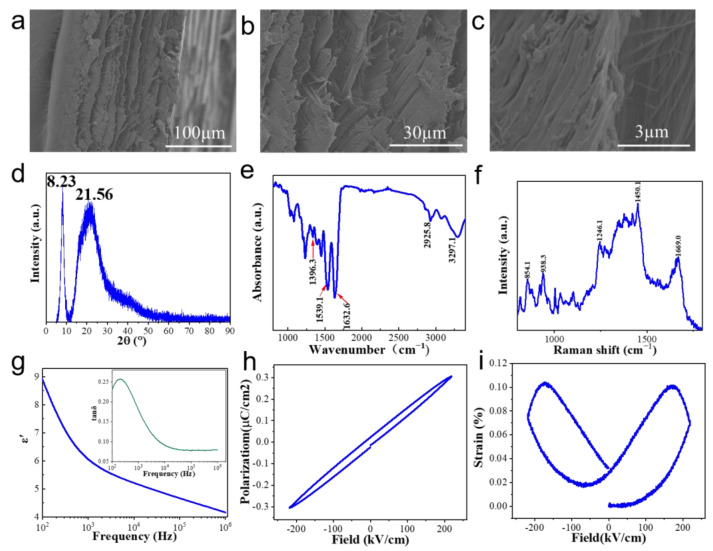
(**a**–**c**) SEM of cross-section images, (**d**) XRD pattern, (**e**) Fourier-transform infrared spectroscopy, (**f**) Raman spectroscopy, (**g**) dielectric constant and loss, (**h**) ferroelectric diagram, and (**i**) electrogenic strain diagram of a demineralized fish scale.

Figure 2a–c show the cross-section SEM images of a demineralized fish scale. It can be clearly seen from this figure that the scale has a multi-layered structure. An enlarged view shown in Figure 2b reveals that each layer is composed of relatively thick fibers. A further enlarged view focusing on one layer displayed in Figure 2c shows that the thick fibers are made up of very smaller fibers. 

**Figure 3:**
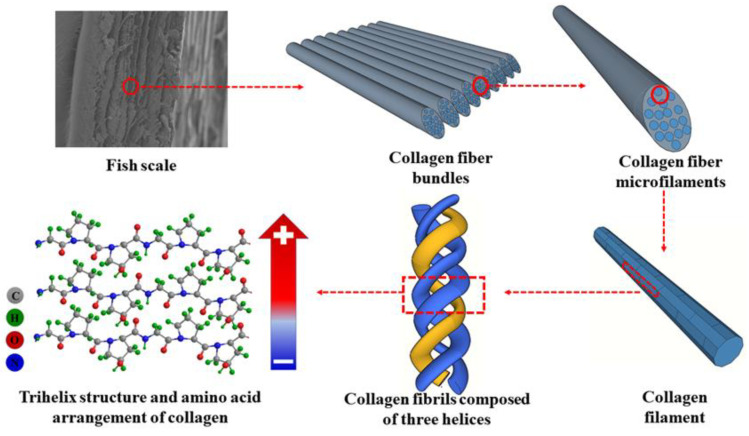
Schematic diagram of fish scale microstructure and the origin of spontaneous polarization as indicated by an arrow.

Figure 2d shows the XRD pattern of the demineralized fish scale. It shows two intensive crystallization peaks located at 2θ = 8.23° and 21.56°. The crystallinity of the fish scale is about 54% deduced by the Fourier deconvolution calculation. The crystals of the fish scale are derived from peptide chains in collagen that rely on a large number of intramolecular and intermolecular hydrogen bonds to form ordered, dense structures. The chemical composition of the fish scale was examined by Fourier infrared and Raman spectra and the results were given in Figure 2e,f, respectively. It can be seen, from the infrared absorption diagram, that there is a characteristic absorption peak at the wave number of 1539.1 cm^−1^. This absorption peak is related to the triple-helix structure, indicating the existence of a triple-helix structure in the fish scales. The obvious absorption peaks at 1336.5, 1632.6, and 3279.1 cm^−1^ are the C-N vibration absorption peak in the amide bond, the carbonyl C=O vibration absorption peak in the amide band, and the N-H vibration absorption peak in the amide band, respectively. This finding reveals that there is an amide structure (-CO-NH-) in the fish scale. The characteristic absorption at wave number 2925.8 cm^−1^ is the C-H vibration absorption peak of the characteristic group R_2_CH_2_ for glycine in amino acid. The Raman peaks at 938.3 and 854.1 cm^−1^ are caused by proline C-C stretching vibration and hydroxyproline C-C stretching vibration, respectively. The 1246.1 cm^−1^ peak represents the n-H vibration characteristics in the amide band. The Raman peak at 1450.1 cm^−1^ is derived from the ethyl (CH_2_) vibration. The Raman peak at 1669.0 cm^−1^ comes from the stretching vibration of amide carbonyl C=O. These results indicate that there is proline, hydroxyproline, and amide structures in the demineralized fish scale. The results of the Raman analysis are in accordance with the FTIR results. Both the FTIR and Raman spectra demonstrate that the fish scale is composed of type I collagen with a triple-helix structure. This type of collagen is known to be made up of three α polypeptide chains. The three polypeptide chains have two identical α_1_ and a single α_2_. Three polypeptide chains are combined in a triple-helix structure to form type I collagen fibrils. Each alpha chain is arranged as follows: Gly-X-Y, with Gly being glycine, and X and Y being proline and hydroxyproline, respectively. The internal structure of the fish scale is schematically shown in Figure 3. The electrical effect between and within the peptide chains in the fish scale mainly comes from the amide carbonyl group in the peptide chain and the hydroxyl group in the hydroxyproline side chain. The arrow shows the generated spontaneous polarization.

Based on the above results, the basic structural units of the fish scale are glycine, proline, and hydroxyproline. The three amino acids are arranged alternately to form a polypeptide chain. The polypeptide chain forms type I collagen fibrils in a triple-helix structure. Collagen fibrils are bonded by hydrogen bonds to form type I collagen microfibrils and type I collagen fiber bundles, and, finally, form a lamellar structure. Spontaneous polarization plays a crucial role in enhancing the surface charge density. To elucidate this point, we test the dielectric, ferroelectric, and electrically induced strain properties of the fish scale. As shown in Figure 2g, the fish scale shows a dielectric constant of 9 at 100 Hz, which gradually decreases with the increase of frequency. The dielectric measurements show that the fish scale has a certain ability to store and release charge. As shown in Figure 2h,i, the scale shows a relatively narrow *P*-*E* loop (Figure 2h) accompanied by an obvious butterfly-type strain loop (Figure 2i), verifying the impressive ferroelectric behavior of the fish scales. The thermal stability of the fabricated TENG device depends on the thermal stability of the fish scale, which was tested by TGA. As shown in Figure S1, when the temperature is increased from 20 to 80 °C, the weight of water in the fish scale begins to disappear. After that, up to 250 °C, the weight loss of the scale is relatively low. However, above 250 °C, the weight of the sample decreases sharply due to the decomposition of the protein at high temperature. At 500 °C, the weight of the sample decreases to 35% of the initial weight. This result shows that the TENG can work in a temperature range lower than 250 °C. 

### 3.2. Fish-Scale TENG Output Performance

**Figure 4:**
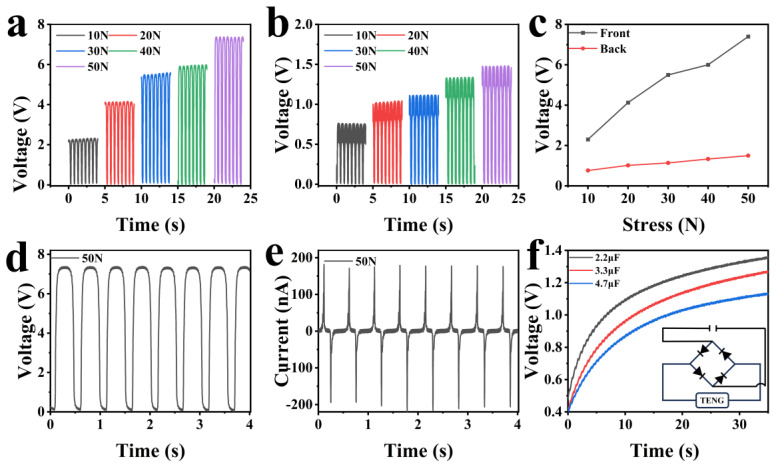
The front (**a**) and back (**b**) output voltage of the fish-scale TENG under different stresses. (**c**) Comparison of the front and back voltages under different pressures. The stability of the output voltage (**d**) and current (**e**) under a constant pressure of 50 N. (**f**) The fish-scale TENG charges different capacitors.

In the case of the contact-separation TENG, the effective contact area of the friction layer plays an important in determining its output performance [48]. Due to the different roughness on both sides of the fish scale, as a friction material, the output performances of both sides are also different. It is necessary to define the front and back sides. Herein, we define the side of the scale in direct contact with the water as the front side, while the side close to the fish’s body is called the back side. In order to test the output performance of the fish-scale TENG, the output voltage and current were measured under constant pressure applied by a linear motor. The results were displayed in Figure 4. It can be seen from Figure 4a,b that both the front and back output voltages of the TENG increase with the increase of applied stress. Figure 4c compares the front and back output voltages as a function of the applied stress. The front output voltage of the TENG increases notably from 2.3 to 7.4 V, while the back output voltage increases generally from 0.75 to 1.5 V, as the pressure is increased from 10 to 50 N. The output voltage on the front side of the fish scale increases faster than that of the back side, because the front side of the fish scale has a larger contact area with the negative electrode material during the contact separation process. The voltage output on the front side of the fish scale exceeds that on its back side due to disparities in the roughness of both sides. Specifically, due to the high projection of the back side of the fish scale in the vertical direction, it is not able to increase the contact area by its own deformation in the actual contact, but, instead, the contact with the FEP is reduced in the actual contact separation process, which, in turn, leads to a decrease in its output.

The long-term stability of a device is crucial for its practical application. The output voltage and current under a pressure of 50 N were repeatedly measured for eight cycles. The results were shown in Figure 4d,e. It is found that the TENG can output a voltage of about 7.4 V and a current of 0.18 µA stably. This fact confirms that the TENG exhibits satisfactory repeatability. Thus, the TENG was used to charge different capacitors through the rectifier bridge shown as an inset in Figure 4f. The TENG can charge the 2.2, 3.3, and 4.7 μF capacitors to 1.0 V in about 6.7, 11.5, and 17 s. This result indicates that the fish-scale TENG can be used as a self-powered device to output signals. In contrast to the work of Singh et al. [31], this work presents applications in harvesting energy from the human body. The notable output voltages yielded by tapping the finger, bending the elbow, and walking provide evidence that our TENG can be used to detect the movements of the human body. Additionally, our TENG exhibits good output performance compared to the other TENGs as highlighted in Table S1. Hence, the TENG shows potential application prospects in healthcare and body information monitoring.

**Figure 5:**
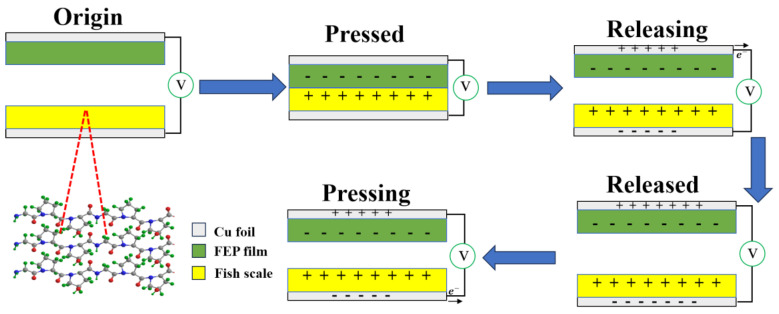
Working principle diagram of the fish-scale TENG.

The power generation mechanism of the fish scale can be understood based on its special structure as illustrated in Figure 5. Because the peptide bonds (-CO-NH-) formed between amino acids and the hydroxyproline side chain form a large number of permanent electrostatic dipoles, a large number of hydrogen bonds are formed between these dipoles and polypeptide chains. The dipoles in the collagen form static polarization by hydrogen bonding when unstressed. The spontaneous polarization of fish scales facilitates the increase of their surface charge density, which, in turn, improves the performance of the fish-scale TENG. When the two materials are separated, an increase in the gap between them triggers electron transfer. Once the separation distance reaches a certain extent, the two electrodes become neutral. When the two materials are in close proximity, electrons are transferred from the fish-scale membrane electrode to the FEP membrane electrode. The TENG consisting of the fish scale/FEP generates current continuously. When the two materials are in full contact, there is no potential difference and they are both in a neutral state.

### 3.3. Applications

**Figure 6:**
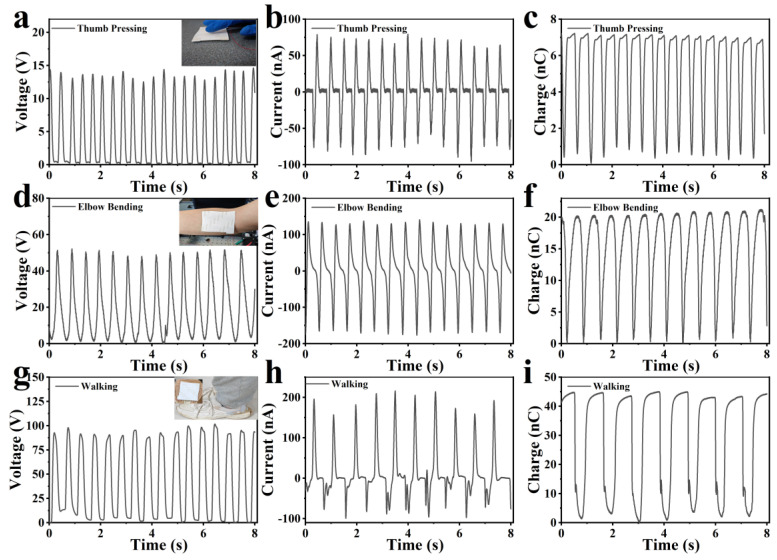
The output signals of the fish-scale TENG used to detect the human activities of thumb pressing (**a**–**c**), elbow bending (**d**–**f**), and walking (**g**–**i**).

The pliability of fish scales enables the fish-scale TENG to function as a wearable and flexible TENG for human activity detection. In light of this, we performed experiments using the TENG to identify instances of walking, finger tapping, and elbow bending. The output signal of the voltage, current, and charge are depicted in Figure 6. Figure 6a–c demonstrate that tapping the TENG with a finger generates a consistent output voltage of 14 V, a current of 75 nA, and a charge of 7 nC. Figure 6d–f show that the TENG can generate stable voltage, current, and charge signals of 50 V, 130 nA, and 20 nC, respectively, when it is used to detect the elbow bending. The results depicted in Figure 6g–i suggest that attaching the TENG to one’s foot while walking promptly elicits a steady voltage signal with a value of approximately 100 V, a current of 200 nA, and a charge of 45 nC. During walking, contact and separation processes occur, resulting in a potential difference between the TENG’s two electrodes and generating a significant voltage signal. A stable voltage output is produced when walking at a constant speed. The data indicate that the voltage generally increases with movement amplitude. Although tapping a finger constitutes a small movement, the TENG still recognizes it and produces the corresponding voltage. The self-powered nanogenerator utilizing fish scales can sensitively detect the movements of the human body for medical monitoring purposes. Once attached to the elbow, the accompanying TENG bends and deforms, thus generating a maximum of 50 V. Therefore, this nanogenerator is highly accurate in its movement detection and generates a significant output signal accordingly. Our results suggest that this TENG holds great promise for large-scale applications in healthcare and body information monitoring. In addition, in order to verify that the TENG can power a device, we charge a 10 μF capacitor, and then use the capacitor to power a calculator. As shown in Figure S2, the TENG charges the capacitor to 2.3 V through the rectifier bridge, which, in turn, is used to power the calculator. 

## 4. Conclusions

In conclusion, we prepared a flexible, wearable, and self-powered triboelectric nanogenerator based on fish scales. The energy-harvesting ability of the TENG was investigated. The output voltage of the TENG increases with an increase in the pressure. The TENG can generate an output voltage of 7.4 V by collecting mechanical energy generated by a constant pressure of 50 N. The TENG can be used to power low-power devices through a rectifier bridge. In addition, as a wearable TENG, it can sensitively detect human activities such as walking, finger tapping, and elbow bending. Our results reveal that the fish-scale TENG holds promising applications in human mechanical energy collection, robot skin, and human body monitoring.

## Data Availability

The data are available upon request.

## Supplementary Materials

The following supporting information can be downloaded at: https://www.mdpi.com/article/10.3390/nano14050463/s1, Figure S1. Thermog—ravimetry analysis of fish scale; Figure S2. Electronic calculator powered by the TENG through the capacitor. Table S1 Comparison of the output properties of fish scales with other triboelectric materials. References [49,50,51] are cited in the supplementary materials.
