# Supplementary material for: Fish Scale for Wearable, Self-Powered TENG
Source: Nanomaterials (Basel). 2024 Mar 3;14(5):463. doi: 10.3390/nano14050463 (PMC10935127; doi:10.3390/nano14050463)
Supplement: Supplementary file 1 [file nanomaterials-14-00463-s001.zip › nanomaterials-2874246-supplementary.pdf]

Supplementary materials

Table of contents

Figure S1. Thermog--ravimetry analysis of Fish scale.
Figure S2. Electronic calculator powered by the TENG through the capacitor.
Table S1. Comparison of the output properties of fish scales with other triboelectric materials.

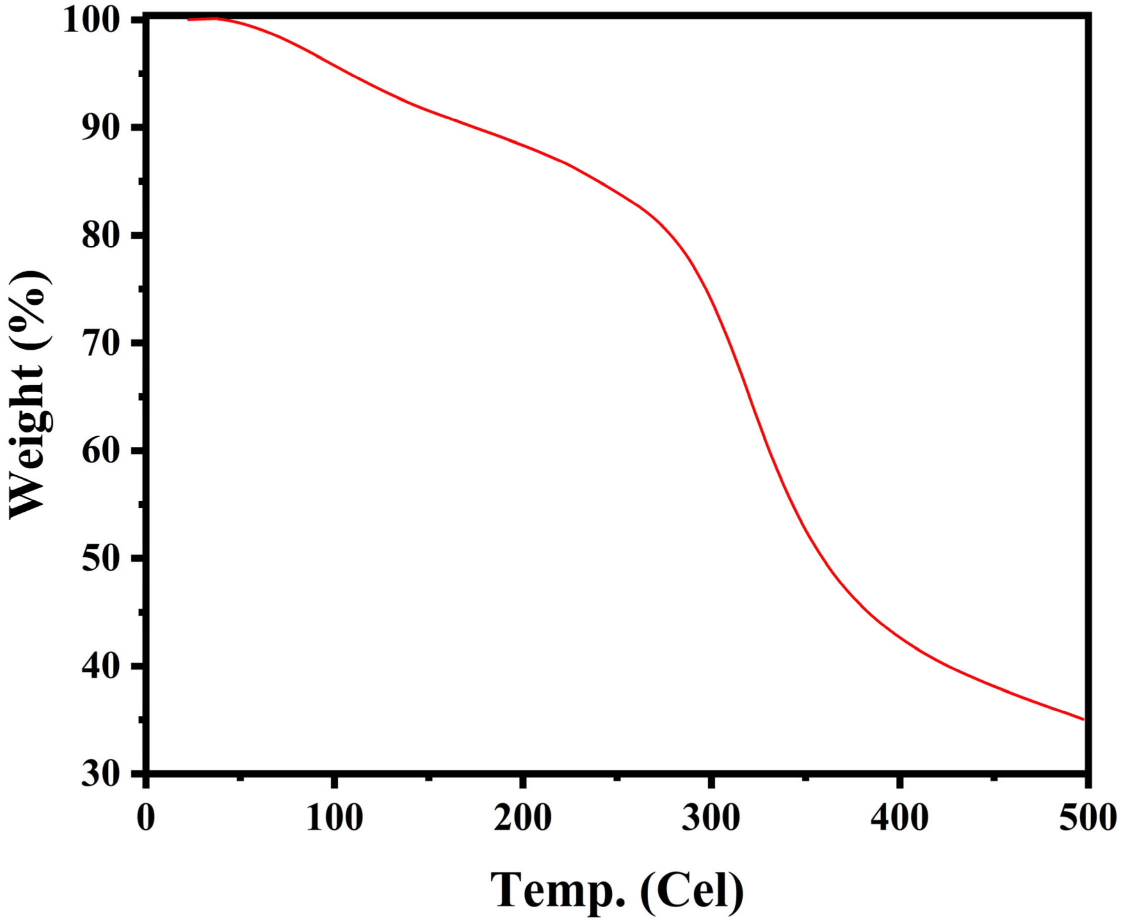

Figure S1. Thermog--ravimetry analysis of fish scale.

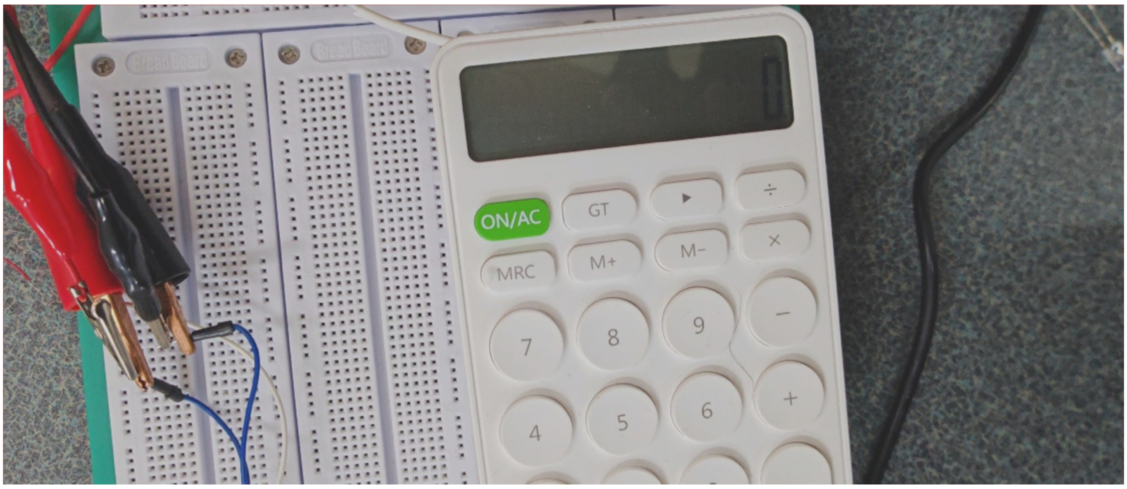

Figure S2. Electronic calculator powered by the TENG through the capacitor.

Table S1. Comparison of the output properties of fish scales with other triboelectric materials.

Material	Voltage (V)	reference
fish scale	7.4	This work
carbon nanotube	25	[49]
ZnO nanosheet	4	[50]
3D knitted spacer fabric	3	[51]
